# Modelling the Progression of Bird Migration with Conditional Autoregressive Models Applied to Ringing Data

**DOI:** 10.1371/journal.pone.0102440

**Published:** 2014-07-21

**Authors:** Roberto Ambrosini, Riccardo Borgoni, Diego Rubolini, Beatrice Sicurella, Wolfgang Fiedler, Franz Bairlein, Stephen R. Baillie, Robert A. Robinson, Jacquie A. Clark, Fernando Spina, Nicola Saino

**Affiliations:** 1 Dipartimento di Biotecnologie e Bioscienze, Università degli Studi di Milano-Bicocca, Milano, Italy; 2 Dipartimento di Economia, Metodi quantitativi e Strategie di Impresa, Università degli Studi di Milano-Bicocca, Milano, Italy; 3 Dipartimento di Bioscienze, Università degli Studi di Milano, Milano, Italy; 4 Max Plank Institute for Ornithology, Vogelwarte Radolfzell, Radolfzell, Germany; 5 Institute of Avian Research “Vogelwarte Helgoland”, Wilhelmshaven, Germany; 6 British Trust for Ornithology, Thetford, United Kingdom; 7 Istituto Superiore per la Protezione e la Ricerca Ambientale, Ozzano dell'Emilia (BO), Italy; University of Lleida, Spain

## Abstract

Migration is a fundamental stage in the life history of several taxa, including birds, and is under strong selective pressure. At present, the only data that may allow for both an assessment of patterns of bird migration and for retrospective analyses of changes in migration timing are the databases of ring recoveries. We used ring recoveries of the Barn Swallow *Hirundo rustica* collected from 1908–2008 in Europe to model the calendar date at which a given proportion of birds is expected to have reached a given geographical area (‘progression of migration’) and to investigate the change in timing of migration over the same areas between three time periods (1908–1969, 1970–1990, 1991–2008). The analyses were conducted using binomial conditional autoregressive (CAR) mixed models. We first concentrated on data from the British Isles and then expanded the models to western Europe and north Africa. We produced maps of the progression of migration that disclosed local patterns of migration consistent with those obtained from the analyses of the movements of ringed individuals. Timing of migration estimated from our model is consistent with data on migration phenology of the Barn Swallow available in the literature, but in some cases it is later than that estimated by data collected at ringing stations, which, however, may not be representative of migration phenology over large geographical areas. The comparison of median migration date estimated over the same geographical area among time periods showed no significant advancement of spring migration over the whole of Europe, but a significant advancement of autumn migration in southern Europe. Our modelling approach can be generalized to any records of ringing date and locality of individuals including those which have not been recovered subsequently, as well as to geo-referenced databases of sightings of migratory individuals.

## Introduction

Migration is widespread in nature and several taxa, from insects to fishes, amphibians, birds and mammals, undertake annual “incredible journeys” that represent key stages in their yearly cycle [1]–[3]. Being able to fly, birds are the taxon where migratoriness is most widespread, and on which the majority of migration studies have focused [2]. As a fundamental feature in the life-history of birds, migration is under strong selective pressures [1]. However, a large amount of genetic variability in migratoriness, timing of migration, and migration strategies exists in bird populations. In addition, individuals show a high degree of phenotypic plasticity in migration strategy [4]. Genetic variability and phenotypic plasticity allow birds to adjust their migration strategies according to changes in climate and ecological conditions. Indeed, changes in the timing (phenology) of migration are considered signals of the impact of current climate changes on the biosphere [5]–[7]. However, many species of migrant birds are declining, probably because they are not able to sufficiently adjust the timing of their annual life-cycle to match new climatic conditions [8]–[10].

Studies of bird migration are hampered by the difficulty of tracking small-sized species, which represent the large majority of migratory birds. New miniaturized and cheap technological devices, like light-level geo-locators, are bridging this gap in our knowledge [11] although the information they provide must be interpreted with caution because of the impact they may have on individual fitness [12]–[14], and, potentially, also on migration timing and routes [12]. Most importantly, these data, together with stable isotope analyses of museum specimens [15], will likely represent, for a long time, the only sources of information allowing for retrospective analyses of changes in bird migration strategies through time.

The analysis of ring recoveries is hampered by several difficulties, the main ones being the large spatial and temporal heterogeneity in ringing effort and in the probability of recovery of a ringed individual [16]. Nevertheless, these data have been useful to study large scale patterns of individual distribution, like migratory connectivity, and long term variation in bird distribution [17]–[20]. Retrospective analyses of ring recoveries may also allow us to quantify both the progression and the timing of migration. *Progression* of migration is defined here as the proportion of individuals of a given migratory population that, at any given time, have reached or have passed over a given place during migration, while migration *timing* is defined here as the date at which a given proportion of individuals have reached a given location.

In the present paper we show how ring recoveries collected throughout Europe and North Africa in 1908–2008 and stored in the EURING databank (EDB, www.euring.org) can be used to model the progression and the timing of migration of bird species, and its variation over time, using a small passerine, the Barn Swallow *Hirundo rustica,* as an example.

Progression and timing of migration can be statistically modelled by fitting the complementary log-log (‘cloglog’ hereafter) function [21], which is very similar to the logistic function (see Text S1), to the cumulated proportion of individuals that have reached a given site by a given date. This modelling approach has the advantage that parameters of the interpolated cloglog curve describe both the progression and the timing of migration at a given site, since the function allows us to estimate both the expected proportion of migrants that have arrived or passed at a given date, and, by model inversion, the date when a given proportion of migrants is expected to have arrived at or passed over a given site.

We first modelled progression of spring migration over the British Isles, taking advantage of the very large amount of data available for this area. We then tentatively extended the same model to Europe and north Africa, where data are sparser. Paucity of data from eastern Europe and the Middle East forced us to restrict the analysis to western Europe and the western part of north Africa (western Europe and north Africa hereafter). The same approach was also used to model autumn migration, first in the data-rich British Isles and then in western Europe and north Africa. Secondly, we modelled the variation of migration phenology over time. To this aim we divided the dataset into three periods containing approximately a similar amount of data (1908–1969, 1970–1990, and 1991–2008) and compared median migration date estimated by the cloglog functions describing progression and phenology of bird migration in different geographical areas. Also in this case analyses were run separately for the British Isles and for western Europe and north Africa and for spring and autumn migration, respectively.

## Materials and Methods

### Datasets

For individually ringed birds, the EDB includes information on date and locality at ringing, as well as at any subsequent encounter. These data will hereafter be defined as “ring recoveries” in order to distinguish them from records of ringing date and locality of individuals, which have not been subsequently recovered (“ringing data” hereafter). Hence ring recoveries include both ringing and finding information of any bird that has been re-encountered.

The EDB almost exclusively include ring recoveries (a few ringing data has been recently included in the EDB, but they were not considered in the present study). Before the analyses we carefully checked the consistency of data in our dataset and excluded any dubious data (details not shown).

The datasets used to model migration in the British Isles consist of 1983 ring recoveries during spring migration (March-June) and 8429 ring recoveries during autumn migration (August-October), while those used for Western Europe and North Africa consist of 11918 ring recoveries (including ring recoveries from the British Isles) collected during spring migration (February-June) and 28832 during autumn migration (August-November) (figure S1). Periods of spring and autumn migration were chosen according to Cramp [22]. Since the relevant information for this analysis is the date at which an individual was observed in a given geographical location, we used all records of individuals found either alive or dead, and retained repeated records of the same individual. Records of nestlings i.e. birds ringed at the nest and unable to fly (EURING age code equal to 1; see Speek *et al.* [23] for further details on the EURING exchange code 2000) were excluded, as well as records of individuals found dead, but not fresh (EURING code ‘condition’ either equal to 1 or 3 [23]) and those of individuals whose recovery date is known with an accuracy larger than 3 days either side of the reported date (EURING code ‘accuracy of date’ in 0–2 [23]). Date of recovery is given in days with January 1 as day 1.

### Conditional autoregressive models

The British Isles were divided in 38 cells of 1.5°×1.5° latitude x longitude, while western Europe and north Africa were divided into 67 4°×4° cells, and each recovery was assigned to a cell (Figure S1). Only cells with ring recoveries recorded in at least four different dates were included in the analyses because interpolation of cloglog curves requires at least four data points per cell. Cell size was chosen as to maximize the geographical coverage of cells suitable for analyses. Only cells to the north of latitude 26° N and to the west of longitude 26° E were considered because data were too scattered outside this area. Some cells could not be included in all analyses due to paucity of data. For the British Isles, 27 cells were included in the spring analyses and 29 in autumn, while for western Europe and north Africa there were 59 cells in spring and 53 in autumn.

The analytical procedure interpolated the cumulated proportion of Barn Swallows recorded in a cell at each date over the periods of spring or autumn migration, by also accounting for the spatial autocorrelation of data recorded at the same time in adjacent cells. Let 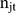 be the cumulated number of individuals observed in cell j until date t, irrespective of the year of recovery, 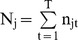 be the total number of Barn Swallows in cell j and 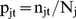 the proportion of Barn Swallows recovered until date t, t = 1, …, T, T being the end of the period of interest (Figure S2). All recoveries were used irrespective of year as data were sparse for some cells. Ordinary binomial regression can be adopted to estimate the cumulative proportion of arrivals in cell j at any given date as a function of a set of secondary variables. We modelled the occurrences in a cell as a linear function of the date on a cloglog scale since this scale is the most appropriate to model spatial point patterns on a geographical grid [21]. To account for spatial autocorrelation and avoid biased estimates, we specified an autobinomial spatial model for arrivals by including among the linear predictors a spatial covariate obtained by calculating the weighted average proportion of Barn Swallows that, at any given date, had reached the cells immediately adjacent to any given cell. To account for potentially different cell counts, each cell in the neighbourhood was weighted by the proportion w_k_  =  N_k_/N_∂j_ of the arrivals at any cell k in the neighbourhood ∂j of cell j out of the global number of arrivals in the neighbourhood, i.e. 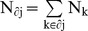.

Cells were considered adjacent when they shared a side or a vertex (‘queen’ configuration [24]). To account for the inter-cell variability in patterns of migration through time, cell identity was entered as a random grouping factor and date as a random slope at the cell level. More formally the model is specified by
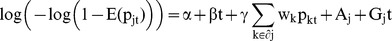
where 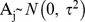 and 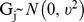, obtaining a Conditional Autoregressive (CAR) binomial Generalized Linear Mixed Model (GLMM).

Note that this model accounts for different numbers of observations at each cell in two ways. Firstly, the dependent variable p_jt_ is specified in the model as the ratio between the cumulative number of individuals that have reached cell j until time t over the total number of individuals in the cell (actually n_it_/(N_j_ – n_it_) in the procedure we used for analyses [25]). The variance of the dependent variable is therefore calculated by taking into account the total number of observations at a cell, thus giving larger weight to cells with more observations. Secondly, the spatial autocovariate is also calculated by giving larger weight to cells in the neighbourhood with more observations.

The ability of the model to correctly interpolate the observed proportion of Barn Swallows that had arrived or had migrated over a given cell in a given date was estimated by calculating a pseudo R^2^ equal to the squared correlation coefficient between observed and estimated cumulated proportions at each cell and date for which there were observations (Efron's pseudo R^2^ for binomial models; [26]). This is a measure of the predictive ability of the model, similar to common R^2^ of linear models, which is undefined for binomial models [27].

These analyses were performed by the *lmer* procedure in the *lme4* package [25] in R 2.15.2 [28]. The cloglog function was interpolated by specifying the cloglog link function in the *lmer* procedure.

### Model inversion and map production

The CAR model was used to predict the exact date at which a given proportion of Barn Swallows had been recovered in a given cell and used to produce contour maps of the date at which a given proportion of Barn Swallows have reached a particular geographical location. In particular, the date t at which a proportion p of swallows are estimated to have reached or have migrated over a given cell j can be calculated as
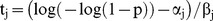
where α_j_ and β_j_ are, respectively, the values of intercept and slope estimated by the CAR binomial GLMM at cell j.

In this paper we produced maps of the calendar date at which 15%, 50% and 85% of Barn Swallows have reached a given cell during spring migration, and maps of the date at which the same percentage of Barn Swallows was still in the cell during the period of autumn migration. In addition, we estimated arrival date of the 5% of Barn Swallows, and date when 5% of Barn Swallows were still in the cell for comparison with arrival dates of the first individual and departure date of the last individual observed at ringing stations or other locations.

Contour maps allow speculations on migration flyways as the contours are isochrones that connect geographical areas showing the same phenology. If Barn Swallows follow flyways during their migration, geographical localities along the flyways might be reached by a given proportion of Barn Swallows earlier in the season than the surrounding areas where the migration movement is less intense. Hence, we expect map contours to show a reverse-U shape in the flyway direction.

### Consistency of observed and model-predicted phenology

We aimed at comparing our model-predicted estimates of migration phenology with known information of phenology derived from the literature. We considered both quantitative estimates of first and mean/median arrival dates or departure dates of the last individual from time series of ringing/observation and qualitative descriptions of migration phenology (Table S1). Quantitative phenological data from time series at a given geographical location were compared to arrival/departure dates estimated by our model for the corresponding percentage and cell.

Qualitative descriptions of migration phenology were also entered in the analysis by converting them to a quantitative estimate (Table S1). We acknowledge that this procedure is based on a subjective interpretation of the qualitative description, but note that excluding these data from the analyses did not alter the results of the following analyses (details not shown).

Consistency and agreement of phenological estimates from our models and observed phenology were assessed by calculating the repeatability [29]; [30] between phenological estimates from the literature and those estimated by our models. Due to paucity of data, we pooled data from both autumn and spring migration. To avoid unduly inflating repeatability due to the (obvious) difference in dates among spring and autumn migration we used the following procedure to centre the data before the analysis. We calculated the mean value of both observed and estimated values for spring migration (common mean for spring migration), and the mean value of both observed and estimated values for autumn migration (common mean from autumn migration). We then subtracted the common mean for spring migration from both observed and estimated values for spring migration, and the common mean for autumn migration from both observed and estimated values for autumn migration.

### Maps of ring recoveries

Maps of ring recoveries assist with the interpretation of movement patterns inferred from contour maps by showing the actual movement of individuals. Information on the movements of individuals is included in ring recoveries, but was not used in the analysis on which contour maps are based. The only information necessary to produce contour maps is indeed the date and the position where an individual has been observed.

Maps of ring recoveries were produced by connecting the positions where an individual was observed, irrespective of the year of recovery. Only records during spring or autumn migration were included. As maps of ring recoveries were used for comparison with contour maps, they only included individuals that moved a range of distances comparable to those that could be inferred from contour maps. For this reason, only individuals that moved between 1 and 8 degrees of latitude or longitude were included in maps of ring recoveries. Indeed, lines connecting the positions of individuals recovered at longer or shorter distances only complicate these maps without providing useful information on patterns of migration.

### Temporal variation in migration phenology

We investigated whether median migration dates varied over time and whether changes in the timing of migration differed among geographical areas [31]. To this end, the dataset was divided into three time periods, 1908–1969, 1970–1990, and 1991–2008. Time limits for these periods were chosen to include a similar amount of data for each period (details not shown). In addition, the British Isles were divided in two latitudinal belts north or south of latitude 53° 45′ N (Figure S1), while western Europe and north Africa were divided into three latitudinal belts (northern Europe: >50° N, central Europe: >42° N & <50° N, southern Europe and north Africa: <42° N; see Figure S1). These thresholds were chosen so as to have at each belt a sufficient number of cells for statistical analyses in each period. For each cell the cumulative proportion of Barn Swallows that had been recovered by a given date within each period was then modelled as a cloglog function of date. The date when the median Barn Swallow was expected to arrive at any cell (‘median migration date’ or t_0_ hereafter) in each period was calculated from the fitted cloglog curve (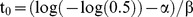 where α and β are, respectively, the values of intercept and slope of the (linearized) cloglog curve fitted at each cell).

Median migration dates in each period and cell were then analysed by using CAR linear mixed models whereby period (three-level factor), belt (two-level factor in the analyses on the British Isles and three-level factor in those on western Europe and north Africa) and their interaction were entered as predictors together with a spatial autocovariate, while cell was entered as a random grouping factor. The value of the spatial autocovariate 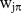 for cell j in each period π was here calculated by averaging median migration dates 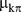 at period π of the 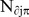 cells in the neighbourhood 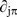 of cell j. Formally:
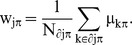



We also corrected our models for heteroscedasticity because graphical exploration showed that variance in median migration dates differed widely between periods. In these models, a significant belt by period interaction would indicate that changes in timing of bird migration between periods differed according to the geographical position of cells.

These analyses were performed with the *lme* procedure in the *nlme* package [32] in R 2.15.2. Models were corrected for heteroscedasticity by specifying an among-period *varIdent* weighting function in the *lme* procedure [32].

## Results

### Maps obtained from CAR models

#### British Isles

**Figure 1:**
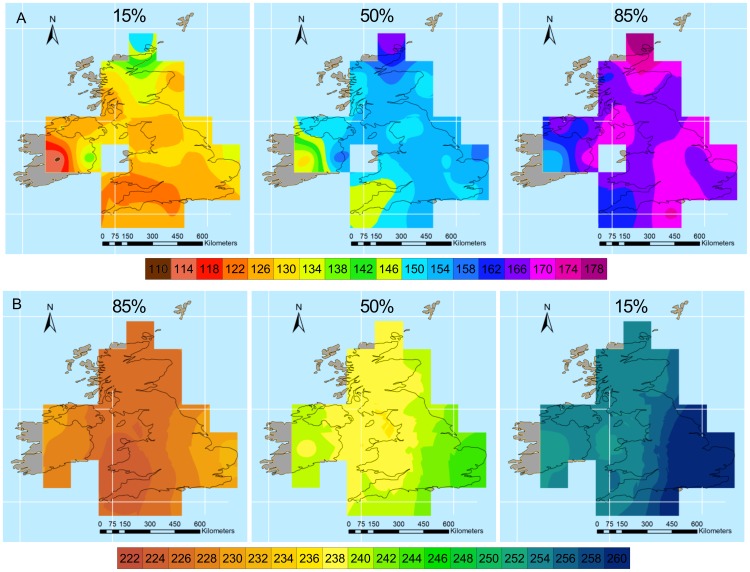
Progression of Barn Swallow migration in the British Isles. Contour plots of the calendar date in which the CAR model predicts that a given percentage of Barn Swallows have been recorded during (A) spring and (B) autumn migration. Contours were generated by linear kriging interpolation. Numbers in the colour scale represent the mean date for each 4-days (spring) or 2-days (autumn) colour belt (1 January  = 1). For ease of interpretation we here report some reference dates: 100 = 31 March, 120 = 30 April; 150 = 30 May, 180 = 29 June, 200 = 19 July, 230 = 18 August, 260 = 17 September.

Maps of spring migration show a general northward progression of migration, but also show some local patterns (Figure 1A). The maps of spring migration in the British Isles indicate that Barn Swallows arrive earlier in central Ireland (estimated migrtion date of the first 15% of Barn Swallows at cell E1 is 109 = 19 April; see Figure S1A for cell IDs) than in the rest of the British Isles (data from southern and western Ireland were unavailable). They then appear to move northwards toward south-west Scotland, where the first 15% of Barn Swallows is expected to pass around 8 May (date  = 128).

South-west England is reached by the first 15% of Barn Swallows at the beginning of May (124 = 4 May), then Barn Swallows seem to move northwards in two main directions, on the one side toward Wales and north-west England, and on the other along the western coast of northern England. Barn Swallows arrive latest in northern Scotland and the Orkney Islands (15% approximately at 140 = 20 May). The bulk of migration (50%) transits in Britain around 30 May (150), with the only exception of central Ireland, where it is earlier (128 = 8 May), and of northern Scotland, where it is later (154 = 3 June). The last Barn Swallows (85%) pass through central Ireland on around 30 May (85% is estimated at date  = 150 at cell E1), then probably move north towards Scotland, which is reached by the last 85% of Barn Swallows on around 13 June (164; Figure 1A).

**Figure 2:**
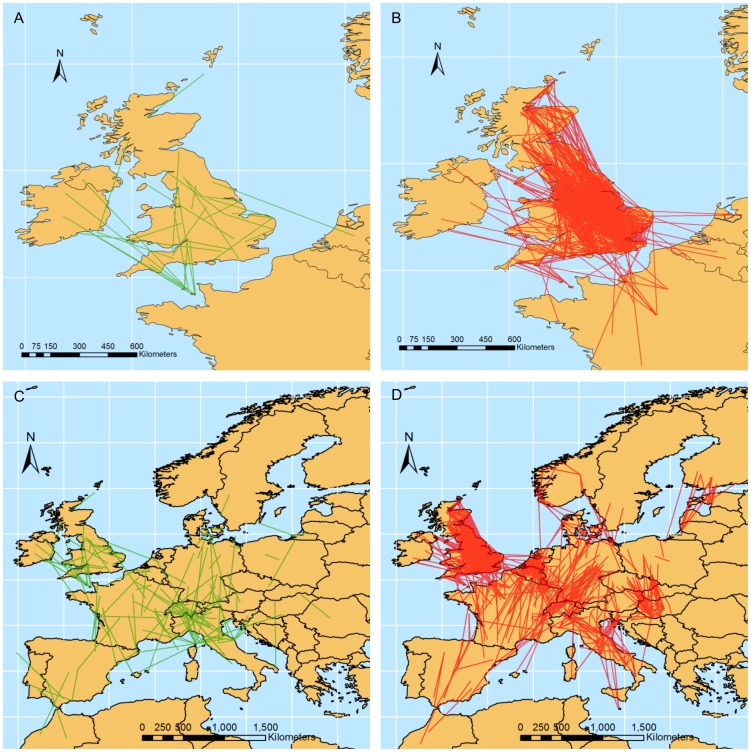
Maps of Barn Swallow movements. Each line connects the ring and recovery position of individual Barn Swallows in (A) March-June and (B) August-October in the British Isles or in (C) February-June and (D) August-November in western Europe and north Africa. To facilitate the interpretation of the figure only Barn Swallows that moved more than 1 and less than 8 degrees latitude or longitude are shown.

The CAR mixed model used to produce the map of spring migration over the British Isles interpolated the observed proportion of Barn Swallows at each cell in each date with great accuracy (R^2^ = 0.95). In addition, the pattern of migration depicted above is consistent with the movements of individual Barn Swallows documented by ring recoveries. Indeed, Figure 2A shows movements of individuals between the Channel Islands and Ireland, thus suggesting direct movements of Barn Swallows towards Ireland.

Maps of autumn migration in the British Isles showed a reverse pattern, with Barn Swallows moving south-east from Wales and Scotland. In addition, Barn Swallows seem to move through the western part of Ireland earlier than from Scotland, and leave eastern England last (Figure 1B). Migratory movements are more synchronous during autumn than spring migration, as indicated by the lower maximum difference in dates represented by isochrones on maps of the autumn compared to spring migration. In addition, autumn migration routes seem to follow a more eastward direction, as suggested by the shape of isochrones which point east in southern England. The predominant eastward movement during autumn migration is confirmed also by the maps of ring recoveries (Figure 2B). More detailed patterns are difficult to assess in this map, probably due to the synchrony of movements during autumn migration. The model used to produce the map interpolated the observed proportion of Barn Swallows at each cell in each calendar date with great accuracy (R^2^ = 0.97).

#### Western Europe and North Africa

**Figure 3:**
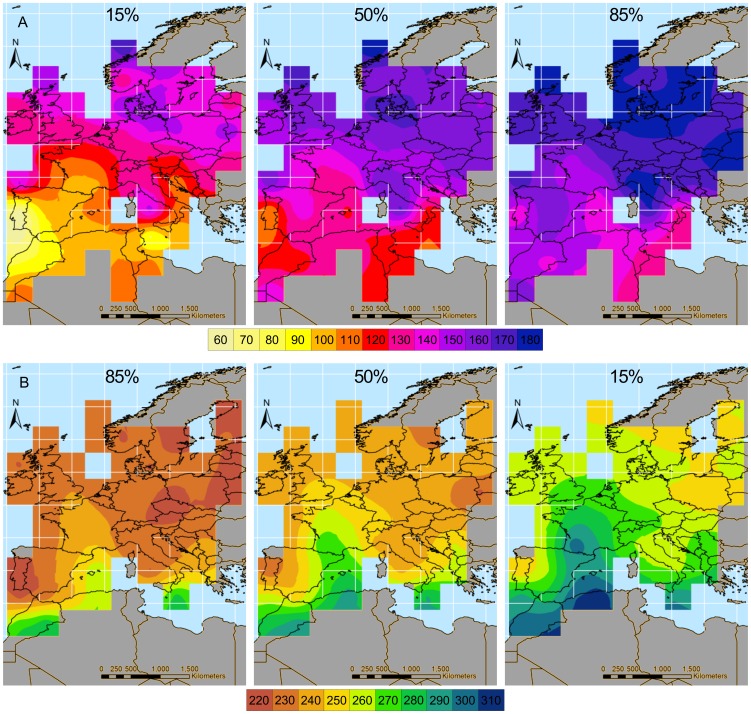
Progression of Barn Swallow migration in western Europe and north Africa. Contour plots of the date in which the CAR model predicts that a given percentage of Barn Swallows have been recorded during (A) spring and (B) autumn migration. Contours were generated by linear kriging interpolation. Numbers in the colour scale represent the mean date for each 10-days colour belt (1 January  =  1). For ease of interpretation we here report some reference dates: 100 = 31 March, 120 = 30 April; 150 = 30 May, 180 = 29 June, 200 = 19 July, 230 = 18 August, 260 = 17 September, 300 = 27 October.

Maps of spring migration over western Europe and north Africa (Figure 3A) were based on a model that interpolated the observed data with great accuracy (R^2^ = 0.96). They show an early transit of birds during spring migration in the Iberian peninsula, with the first 15% of Barn Swallows in southern Portugal on 1 March (60) and in central Spain and southern France on 10 April (100). They seem then to spread north-eastwards in France and the rest of northern Europe, reaching southern Sweden on 20 May (140).

The first 15% of Barn Swallows reaches southern Italy at the beginning of April, then they move towards the Balkans. Interestingly, the maps of the 15%, 50% and 85% of Barn Swallows suggest a progressive eastward shift of the northward turn of isochrones from Spain towards the Balearic Islands, thus suggesting that late migrants may embark in a more direct cross of Mediterranean than early migrants (Figure 3A).

The maps of autumn migration were also based on a model that fitted the data with great accuracy (R^2^ = 0.97). They indicate that the first 15% of Barn Swallows has already crossed Gibraltar on 18 August (230; Figure 3B). A large migration divide seems to occur in France, with Barn Swallows moving along two main migration routes, one along the Atlantic coast toward Spain and Gibraltar, and the other across Switzerland and along the Italian peninsula, with a possible crossing of the Mediterranean from central Italy toward Tunisia, thus embarking on a direct Mediterranean crossing [33]. A map of the last 15% of migrants also suggests that Barn Swallows from north-eastern Europe may move westwards across the Balkans and reach central and southern Italy, although paucity of data from Eastern Europe prevented a clear assessment of movement patterns in this area.

Phenology estimated by our model was generally consistent with that observed in different areas of Europe, as indicated by the significant repeatability among observed arrival/departure dates and those estimated by our model (Table S1, R = 0.43±0.16 SE, F_28,27_ = 2.51, P = 0.009). Repeatability analyses conduced on spring and autumn data separately showed a significant repeatability between observed and estimated phenology for spring migration (R = 0.55±0.16 SE, F_18,19_ = 3.49, P = 0.005), and a non-significant repeatability for autumn migration (R = 0.25±0.33 SE, F_8,9_ = 1.68, P = 0.227).

### Temporal variation in migration phenology

CAR mixed models restricted to the British Isles did not show any significant variations in median date of spring migration, either according to period (Likelihood Ratio Test: χ^2^_2_ = 3.19, P = 0.203), latitudinal belt (χ^2^_1_ = 1.61, P = 0.205), or their interaction (χ^2^_2_ = 1.36, P = 0.507, details not shown). Median autumn migration date in the British Isles did not change significantly between periods (χ^2^_2_ = 0.52, P = 0.770), latitudinal belt (χ^2^_1_ = 0.26, P = 0.607) or their interaction (χ^2^_2_ = 1.06, P = 0.590, details not shown).

**Figure 4:**
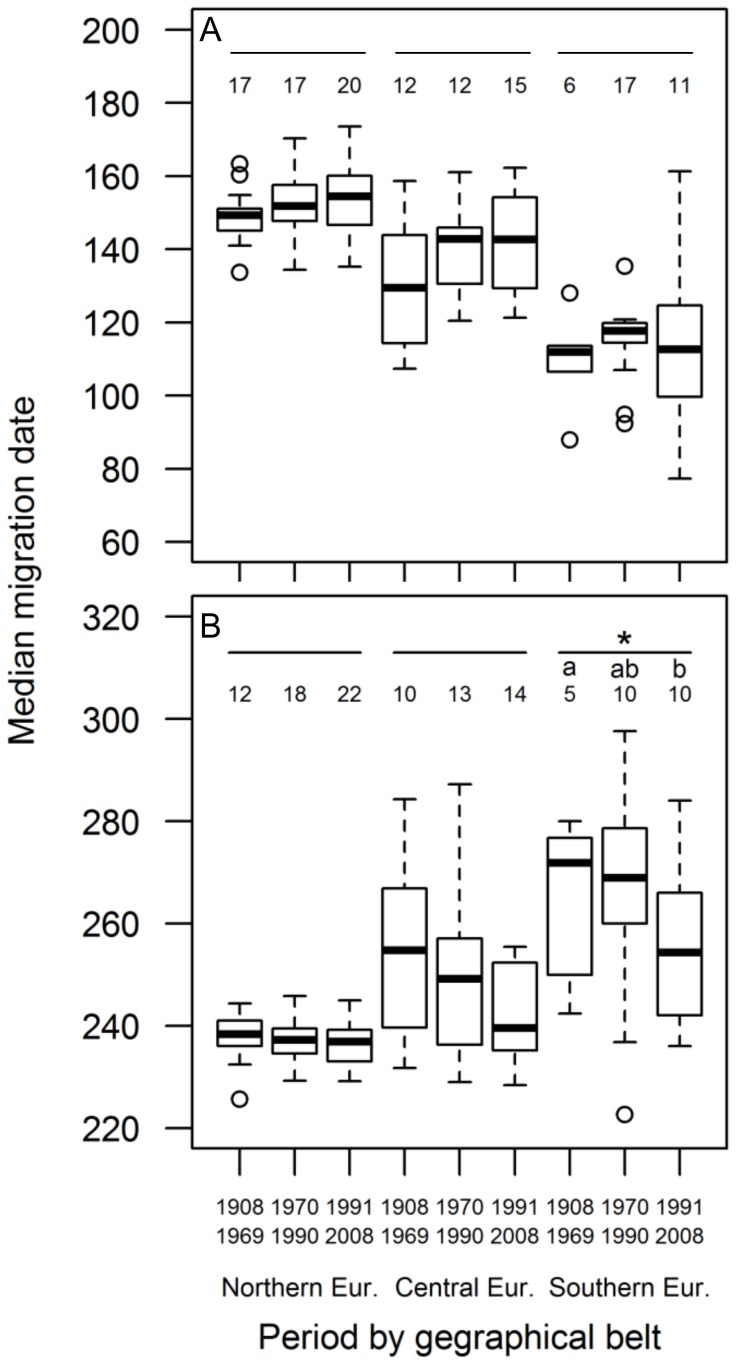
Boxplot of median A) spring and B) autumn migration dates in western Europe and north Africa. Dates were estimated at each cell (t_0_ parameter of cloglog curves interpolated at each cell) in all belt-by-period combinations. The solid line represent the median value, the top and the bottom of the boxes represent the first and the third quartile while whiskers approximately include 95% of data. Circles represent outliers. Numbers represent sample size (i.e. number of cells per period and belt). Asterisk denotes the belt that differs significantly from the others at Tukey post-hoc tests (z≤-3.212, P≤0.004 in all cases). Different letters denote periods that differ significantly to each other within each latitudinal belt at Tuckey post-hoc tests (z = 3.612, P = 0.001).

Median spring migration date changed significantly between latitudinal belts in western Europe and north Africa (χ^2^_2_ = 21.55, P<0.001), but not between periods (χ^2^_2_ = 2.07, P = 0.355). The belt by period interaction was non-significant (χ^2^_4_ = 3.43, P = 0.488; Figure 4A).

The model fitted to autumn data indicated that the belt by period interaction was significant (χ^2^_4_ = 11.81, P = 0.019), as well as the main effect of belt (χ^2^_2_ = 20.34, P<0.001). Post-hoc tests showed that migration was later in southern than in central and northern Europe (z≤−3.21, P≤0.004) and that in southern Europe autumn migration post-1990 was 13.36±3.80 SE days earlier than pre-1970. There were no significant differences in the timing of autumn migration in central and northern Europe (Figure 4B).

## Discussion

In this study we analysed ring recoveries spanning from 1908 to 2008 to describe patterns of bird migration and their long-term temporal trends. A first set of analyses was based on a subset of ring recoveries in the British Isles, and a second set tentatively extended the analyses to Western Europe and North Africa, where data are sparser.

We produced maps describing both spring and autumn migration phenology over the British Isles and, tentatively, over western Europe and north Africa, from which the main migration flyways could be inferred. We found no significant changes in migration phenology of the Barn Swallows in the British Isles, but an earlier timing of autumn migration in southern Europe and north Africa in 1991–2008 compared to 1908–1969. No change in autumn migration phenology was observed in central and northern Europe as well as in spring migration phenology over the whole western Europe and north Africa.

Ring recoveries currently represent the largest and only long-term datasets on bird migration, yet they are hampered by several potential sources of bias, primarily due to large spatial and temporal variation in sampling effort. Previous studies that faced the same problem tried to account for these potential sources of bias, by re-running the analyses on different subsets of data or by including additional variables (see e.g. [18]; [19]). These approaches could not be applied in this case, because this study focuses on migration periods only, and therefore analyses cannot be restricted to ‘focal’ periods of migration without losing important information on early and late migrants, which are relevant for modelling the progression of migration in a given area correctly. In addition, results from analyses accounting for other potential sampling biases may be difficult to interpret. For example, recovery condition of individuals, indicating, for instance, whether an individual was actively trapped or fortuitously recovered, and whether a bird was dead or alive at the time of recovery, were included in previous analyses of ringing recoveries [18]. However, trapped birds may provide early- or late-biased estimates of the timing of bird migration depending on the scheduled activities of ringing stations, which may vary between years and geographical regions. Recoveries of dead or live birds may also show different biases. For example, if birds are more likely to be found dead early than late in spring, and late than early in autumn, analyses restricted to birds found alive or dead may bias the outcome in opposite directions. In addition, analyses restricted to subsets of data may be more prone to produce biased results due to lower power of analyses based on reduced sample size than the whole dataset, which will give the most robust and the least biased results. All the analyses presented in this study therefore used the whole dataset, and all the results should be considered by keeping in mind that it was impossible to conduct additional analyses accounting for possible sources of bias.

Phenological estimates from our models were generally consistent with phenology of the spring and autumn migration described by both quantitative and qualitative observations in literature. We acknowledge that on this analysis we were forced by the scarcity of the data to cumulate information from spring and autumn migration as well as from quantitative and qualitative observations. However, repeatability of observed (from direct observation or trapping of live birds) and estimated (from our models) values was significant, thus confirming the general consistency of our estimates and the observed phenology. Closer observation of the data reported in Table S1, however, shows some differences between observed and estimated phenology. Comparison with time series of first arrival dates indicates that our model estimated that the first 5% of Barn Swallows arrive 1 (Norfolk, England [34]) to 31 days later (Leicestershire, England [35]) than the first swallow was observed. Arrival time of the first 5% of Barn Swallows should be close to but later than that of first observations. Time shifts of 21–31 days between our estimates and observation at sites like Parchim (Northern Germany [36]), Brescia (Northern Italy, [37]), or in Leicestershire (Table S1) are therefore not negligible, even if we consider that the first Barn Swallows usually arrive much earlier than the bulk of migration [38].

Estimates of median arrival dates from our model were consistent with those of the only published time series of mean/median arrival date that was available to us (Ventotene, Southern Italy [39]; Table S1). In addition, we were able to reconstruct arrival dates of 15%, 50% and 85% of Barn Swallows in Kraghede (Denmark) [40]. In this case our model estimated phenology about one month later than that observed (Table S1), suggesting that our results may depict a somewhat later phenology than site-specific time series, at least at some geographical areas. This later estimate of timing of migration provided by our results may therefore suggest that early migrants are underrepresented in ring-recoveries, that our method underestimates the proportion of early migrants, or both. In addition, spring migration spans several months and some records may refer to Barn Swallows captured at breeding sites some times after that they have arrived. At the same time, it is questionable whether time series of arrival at single, selected localities can reliably reflect arrival dates at areas as large as four degrees latitude per longitude. This latter interpretation is suggested by the consideration that time series of arrival dates are usually collected at ringing stations, which are located at key places along migration routes, and at localities that may be close to the margins of a cell. However, our estimates are consistent with the general, qualitative description of migration phenology over larger geographical areas (Table S1).

Comparisons of results about the timing of autumn migration are more problematic, since information of autumn migration phenology is sparser than that on spring migration. Published information from Spain [41] depicts an earlier departure of Barn Swallows than that estimated by our model. However, our model estimated that the last 5% of Barn Swallows are still in central Spain (cell G2, Figure S1D) on 25 September (268), a date very close to that of 21 September reported by Gordo & Sanz [41] as the mean departure date of the last Barn Swallow from Spain. Mean autumn passage date of Barn Swallows at the Col de Bretolet (Switzerland) is 19 days later than median passage date estimated by our model (Table S1). Similarly, departure date of the last Barn Swallow at four ornithological observatories in the UK is 30 to 54 days later than the date at which only 5% of Barn Swallows are still in the area estimated by our models. Conversely, mean departure dates of Barn Swallows from northern Italy based on a short (15 years) unpublished time series (R. Ambrosini, unpublished data) are earlier than the date estimated by our model for the presence of the last 5% of Barn Swallows in the cell (Table S1). In conclusion, evidence of the ability of our model to accurately estimate autumn migration phenology is not unequivocal, but information on the timing of departure of ‘extreme’ individuals at selected localities may not properly represent the general timing of migration over larger geographical areas.

Different independent sources of information however confirmed the reliability of our modelling approach. The pattern of spring migration depicted in the maps of the British Isles we produced is qualitatively consistent with the south-west to north-east pattern of Barn Swallow migration progression through Britain described by Huin & Sparks [42] by using phenological records compiled before 1947. The fact that our maps indicate a migration phenology about 20 days later than that described by Huin & Sparks (e.g. 124 to 128 = 6 to 8 May in central England in our map vs. 109 = 19 April in Huin & Sparks [42]) can be explained by considering that the maps by Huin & Sparks are based on first observation dates while ours are based on the 15% of migration movements. In addition both in our maps and in the paper by Huin & Sparks the time-difference between isochrones though Britain is 20 days (Figure 1A and [42]). Hence, despite the difference in the timing of migration, both studies consistently indicated that Barn Swallows arrive in northern Scotland about 20 days later than in southern England (approximately 800 km to the south).

The second main aim of the present study was to investigate variation over time in timing of migration. No significant change in spring and autumn phenology in the British Isles was detected, which is consistent with Mason [43], who did not find a long-term trend in arrival dates of Barn Swallows between 1942 and 1991, and with Sparks & Carey [34], who found only a slight trend toward a later arrival over two centuries.

No significant change in timing of spring migration appeared neither in the analyses on western Europe and north Africa. Barn Swallows are known to have advanced first arrival dates throughout Europe. For example, Barn Swallows advanced first arrival date by 13 days in 1970–2004 in the Iberian peninsula [41], and mean/median arrival dates advanced by 0.34 days year^−1^ in 1982–2006 in northern Italy [37] and by 0.17 days year^−1^ in 1960–2006 in Europe [8]. Our model was therefore unable to capture this widespread advancement, probably because the paucity of data from ring recoveries forced us to calculate median arrival dates over periods as long as 20 years or more and because of a large heterogeneity among periods in the variance of median arrival dates in cells (despite accounting for this problem in the statistical analyses; see Methods). However, arrival dates of migrant birds, and of Barn Swallows in particular, may have varied non-linearly over the study period [41]. Indeed, Barn Swallows in Spain delayed their arrival dates during the seventies and then have advanced, reaching the same arrival dates as pre-1970 only in recent years. Our analyses are partly consistent with this pattern. Median spring arrival dates seem to have been delayed in 1970–1990 in southern Europe and have then advanced, returning to pre-1970 levels in the last decades (Figure 3A). In addition, cloglog curves interpolating arrival dates in central Spain (cell G2 of Figure S1) pre-1970 and post-1990 almost overlap, while that for 1970–1990 was shifted towards later arrivals (Figure S3).

Parameters of the cloglog curves indicate a significant advancement in autumn migration in southern Europe and north Africa (Figure 4B). This pattern is consistent with that found by Gordo & Sanz [41], who documented an advancement in autumn migration in the Iberian Peninsula. In addition, Jenni and Kéry [44] reported a delay in mean autumn migration at the Col de Bretolet in 1970–1982 with respect to earlier years, and a subsequent advancement. Also this pattern is consistent with the results of our model, that estimated a delay of 6 days in median migration date at the cell including this Swiss locality (E5, see Figure S1) between 1970–1990 and pre-1970, and a subsequent advancement of 11 days between 1970–1990 and post-1990 (other details not shown).

In summary, in this study we propose a novel method to describe patterns of migrations and main routes followed by migratory birds based on ring recoveries. Importantly, this method does not use information on the movements of individuals between locations where they were observed at different times of their lives, but is entirely based on the information on the date at which a bird has been observed in a given place. It may therefore be possible to extend its application to other, potentially larger, datasets. For example, ringing data, which are by far much more abundant than ring recoveries, can be used for this purpose. The main disadvantage is that – so far – only a few of these data are stored in the EDB. They are therefore more difficult to access for continent-wide analyses, and they are more prone to temporal sampling biases (e.g. non-random variation in sampling effort both within and between seasons; [15]). However, they may allow detailed studies at the scale of smaller geographical areas (e.g. countries). Similarly, this method may be applied to sighting databases, such as those collected via the web (e.g. BTO BirdTrack project http://blx1.bto.org/birdtrack; the ORNITHO family portals e.g. www.ornitho.ch), which are becoming increasingly popular in recent years, and to databases of timing of flowering and leafing [45].

Ring recoveries and museum specimens provide the only available data spanning over long time periods, and thus they are the only data allowing investigation of the variation over time of migration phenology over large geographical areas. If ringing data too were available over long time periods, the increased amount of data available for the analyses may allow the use of reduced intervals so that more detailed variation in migration phenology over time can be explored.

## Supporting Information

Figure S1 **Maps of ring recoveries of Barn Swallows.** Exact position of ring recoveries of Barn Swallows in (A) March-June and (B) August-October in the British Isles and in (C) February-June and (D) August-November in western Europe and north Africa. 1908–1969: green; 1970–1990: blue, 1991–2008: red. Cell ID is shown. Parallels separating latitudinal belts are shown.
(TIFF)

Figure S2 **Example of data interpolation.** Complementary log-log curve was interpolated to the March-June data from cell E1 in the British Isles (see Figure S1). Dots represent cumulated proportion of Barn Swallows recovered in this cell at different dates. All data in 1908–2008 were used. The dashed line represents June 30.
(TIFF)

Figure S3 **Interpolated curves showing shifts in migration phenology among periods.** Complementary log-log curves were interpolated to cumulated proportions of Barn Swallows recovered during (A) February-June and (B) August-November during three different periods at cell G2 in western Europe and north Africa (Spain, see Figure S1). Green: curve fitted to data in 1908–1969; blue: curve fitted to data in 1970–1990; red: curve fitted to data in 1991–2008. In A) the delay in spring migration timing in 1970–1990 is evident as well as similarity in spring migration phenology pre-1970 and post-1990. Curves in (B) evidence the advancement in autumn migration phenology post-1970. The dashed line in (A) represents June 30 while that in (B) represents August 1.
(TIFF)

Table S1 **Comparison of observed and estimated phenology.** General description of timing of migration and quantitative information from time-series of arrival dates of Barn Swallows in the British Isles and in western Europe and north Africa were collected from the literature, websites or other unpublished datasets, and compared with the corresponding estimate from our models.
(PDF)

Text S1 **The complementary log-log and logistic functions.** A brief description of complementary log-log and logistic functions.
(PDF)

## References

[1] Newton I (2008) The Migration Ecology of Birds. London: Academic Press. 976 p.

[2] Bauer S, Barta Z, Ens BJ, Hays GC, McNamara JM, et al (2009) Animal migration: linking models and data beyond taxonomic limits. Biol Lett 5: 433–435. 19465574 10.1098/rsbl.2009.0324 PMC2781941

[3] Bauer S, Hoye B (2014) Migratory animals couple biodiversity and ecosystem functioning worldwide. Science 344: 1242552. 24700862 10.1126/science.1242552

[4] Delmore KE, Fox JW, Irwin DE (2012) Dramatic intraspecific differences in migratory routes, stopover sites and wintering areas, revealed using light-level geolocators. Proc R Soc Lond B 279: 4582–4589. 10.1098/rspb.2012.1229 PMC3479719 23015629

[5] Walther G, Post E, Convey P, Menzel A, Parmesan C, et al (2002) Ecological responses to recent climate change. Nature 416: 389–395. 11919621 10.1038/416389a

[6] Parmesan C, Yohe G (2003) A globally coherent fingerprint of climate change impacts across natural systems. Nature 421: 37–42. 12511946 10.1038/nature01286

[7] Parmesan C (2006) Ecological and evolutionary responses to recent climate change. Ann Rev Ecol Syst 37: 637–669.

[8] Møller AP, Rubolini D, Lehikoinen E (2008) Populations of migratory bird species that did not show a phenological response to climate change are declining. Proc Natl Acad Sci USA 105: 16195–16200. 18849475 10.1073/pnas.0803825105 PMC2571031

[9] Robinson RA, Crick HQP, Learmonth JA, Maclean IMD, Thomas CD, et al (2009) Travelling through a warming world: climate change and migratory species. Endang Species Res 7: 87–99.

[10] Sanderson FJ, Donald PF, Pain DJ, Burfield IJ, van Bommel FPJ (2006) Long-term population declines in Afro-Palearctic migrant birds. Biol Cons 131: 93–105.

[11] Stutchbury BJM, Tarof SA, Done T, Gow E, Kramer PM, et al (2009) Tracking long-distance songbird migration by using geolocators. Science 323: 896–896. 19213909 10.1126/science.1166664

[12] Costantini D, Møller AP (2013) A meta-analysis of the effects of geolocator application on birds. Curr Zool 59: 697–706.

[13] Arlt D, Low M, Pärt T (2013) Effect of geolocators on migration and subsequent breeding performance of a long-distance passerine migrant. PLoS ONE 8: e82316. 24324770 10.1371/journal.pone.0082316 PMC3852741

[14] Scandolara C, Ambrosini R, Caprioli M, Hahn S, Lardelli R, et al. (2014) Impact of miniaturized geolocators on the barn swallow (*Hirundo rustica*). J Avian Biol (in press).

[15] Møller AP, Fiedler W (2010) Long-term time series of ornithological data. In: Møller AP, Fiedler W, Berthold P, editors. Effects of climate change on birds. Oxford: Oxford University Press. pp. 33–38.

[16] Korner-Nievergelt F, Sauter A, Atkinson PW, Guélat J, Kania W, et al (2010) Improving the analysis of movement data from marked individuals through explicit estimation of observer heterogeneity. J Avian Biol 41: 8–17.

[17] Fiedler W (2003) Recent changes in migratory behaviour of birds: a compilation of field observations and ringing data. In: Bertholds P, Gwinner E, Sonnenschein E, editors. Avian migration. Berlin: Springer. pp. 21–38.

[18] Ambrosini R, Rubolini D, Møller AP, Bani L, Clark J, et al (2011) Climate change and the long-term northward shift in the African wintering range of barn swallows *Hirundo rustica* . Clim Res 49: 131–141.

[19] Ambrosini R, Møller AP, Saino N (2009) A quantitative measure of migratory connectivity. J Theor Biol 257: 203–211. 19108778 10.1016/j.jtbi.2008.11.019

[20] Visser ME, Perdeck AC, van Balen JH, Both C (2009) Climate change leads to decreasing bird migration distances. Glob Change Biol 15: 1859–1865.

[21] Baddeley A, Berman M, Fisher NI, Hardegen A, Milner RK, et al (2010) Spatial logistic regression and change-of-support in Poisson point processes. Electron J Statist 4: 1151–1201.

[22] Cramp J (1988) Handbook of the Birds of Europe, the Middle East and North Africa. The birds of the Western Palearctic Vol. V. Oxford: Oxford University Press. 1063 p.

[23] Speek G, Clark JA, Rohde Z, Wassenaar RD, Van Noordwijk AJ (2001) The EURING Exchange-Code 2000. Heteren: Vogeltrekstation Arnhem. 147 p.

[24] Bivand RS, Pebesma EJ, Gómez-Rubio V (2008) Applied Spatial Data Analysis with R. New York: Springer. 378 p.

[25] Bates D, Maechler M, Bolker B (2012) *lme4*: Linear mixed-effects models using S4 classes. R package version 0.999999-0. 74 p.

[26] Efron B (1978) Regression and ANOVA with zero-one data: Measures of residual variation. J Am Stat As 73: 113–121.

[27] Nagelkerke NJD (1991) A note on a general definition of the coefficient of determination. Biometrika 78: 691–692.

[28] R Core Team (2012) R: A Language and Environment for Statistical Computing. Vienna: R Foundation for Statistical Computing. 1731 p.

[29] Lessells CM, Boag PT (1987) Unrepeatable repeatabilities, a common mistake. Auk 104: 116–121.

[30] Nakagawa S, Schielzeth H (2010) Repeatability for Gaussian and non-Gaussian data: a practical guide for biologists. Biol Rev 85: 935–956. 20569253 10.1111/j.1469-185X.2010.00141.x

[31] Rubolini D, Møller AP, Rainio K, Lehikoinen E (2007) Intraspecific consistency and geographic variability in temporal trends of spring migration phenology among European bird species. Clim Res 35: 135–146.

[32] Pinheiro J, Bates D, DebRoy S, Sarkar D, the R Core team (2008) *nlme*: Linear and Nonlinear Mixed Effects Models. R package version 3.1–89. 336 p.

[33] Rubolini D, Gardiazabal Pastor A, Pilastro A, Spina F (2002) Ecological barriers shaping fuel stores in barn swallows *Hirundo rustica* following the central and western Mediterranean flyways. J Avian Biol 33: 15–22.

[34] Sparks TH, Carey PD (1995) The response of species to climate over two centuries: an analysis of the Marsham phenological record, 1736–1947. J Ecol 83: 321–329.

[35] Mackay AJ (2002) The Leicestershire and Rutland Bird Report 2001. Leicester: The Leicestershire and Rutland Ornithological Society.

[36] Schmidt E, Hüppop K (2007) First observation and start of birdsong of 97 bird species in a community in the county of Parchim (Mecklenburg-Vorpommern) in the years 1963 to 2006. Vogelwarte 45: 27–58.

[37] Rubolini D, Ambrosini R, Caffi M, Brichetti P, Armiraglio S, et al (2007) Long-term trends in first arrival and first egg laying dates of some migrant and resident bird species in northern Italy. Int J Biometeorol 51: 553–563. 17375338 10.1007/s00484-007-0094-7

[38] Turner A (2006) The Barn Swallow. London: T & A D Poyser. 256 p.

[39] Spina F, Massi A, Montemaggiori A (1994) Back from Arica: who's running ahead? Differential migration of sex and age classes in Palearctic-African spring migrants. Ostrich 65: 137–150.

[40] Møller AP (1994) Phenotype-dependent arrival time and its consequences in a migratory bird. Behav Ecol Sociobiol 35: 115–122.

[41] Gordo O, Sanz JJ (2006) Climate change and bird phenology: a long-term study in the Iberian Peninsula. Glob Change Biol 12: 1993–2004.

[42] Huin N, Sparks TH (1998) Arrival and progression of the Swallow *Hirundo rustica* through Britain. Bird Study 45: 361–370.

[43] Mason CF (1995) Long-term trends in the arrival dates of spring migrants. Bird Study 42: 182–189.

[44] Jenni L, Kéry M (2003) Timing of autumn bird migration under climate change: advances in long-distance migrants, delays in short-distance migrants. Proc R Soc Lond B 270: 1467–1471. 10.1098/rspb.2003.2394 PMC1691393 12965011

[45] Schwartz MD (2003) Phenology: An Integrative Environmental Science. Dordrecht: Kluver Academic Publisher. 571 p.

