# Supplementary figures and images for: Modelling the Progression of Bird Migration with Conditional Autoregressive Models Applied to Ringing Data
Source: PLoS One. 2014 Jul 21;9(7):e102440. doi: 10.1371/journal.pone.0102440 (PMC4105499; doi:10.1371/journal.pone.0102440)

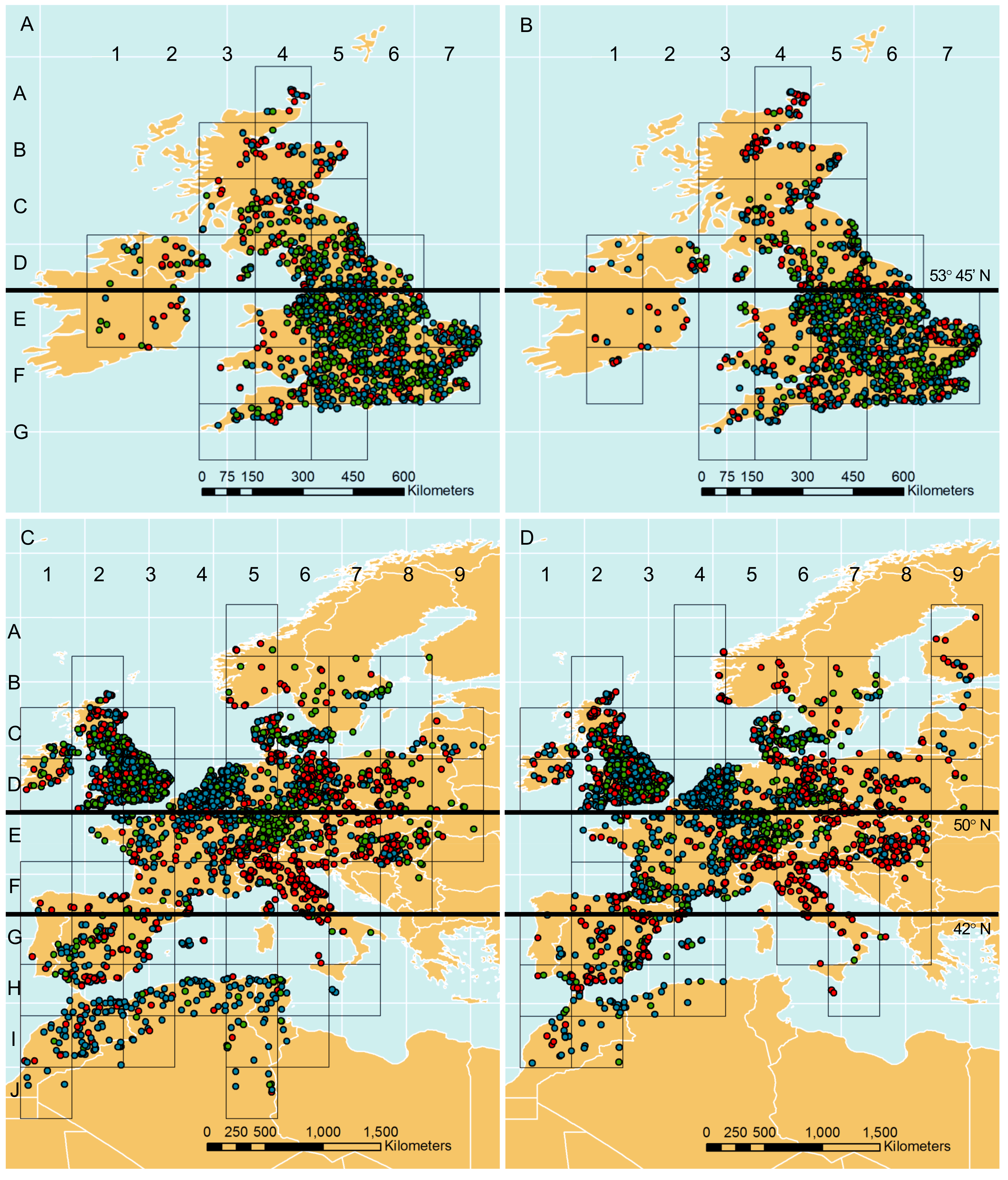

Supplement: Figure S1 — Maps of ring recoveries of Barn Swallows. Exact position of ring recoveries of Barn Swallows in (A) March-June and (B) August-October in the British Isles and in (C) February-June and (D) August-November in western Europe and north Africa. 1908–1969: green; 1970–1990: blue, 1991–2008: red. Cell ID is shown. Parallels separating latitudinal belts are shown. (TIFF) [file pone.0102440.s001.tiff]

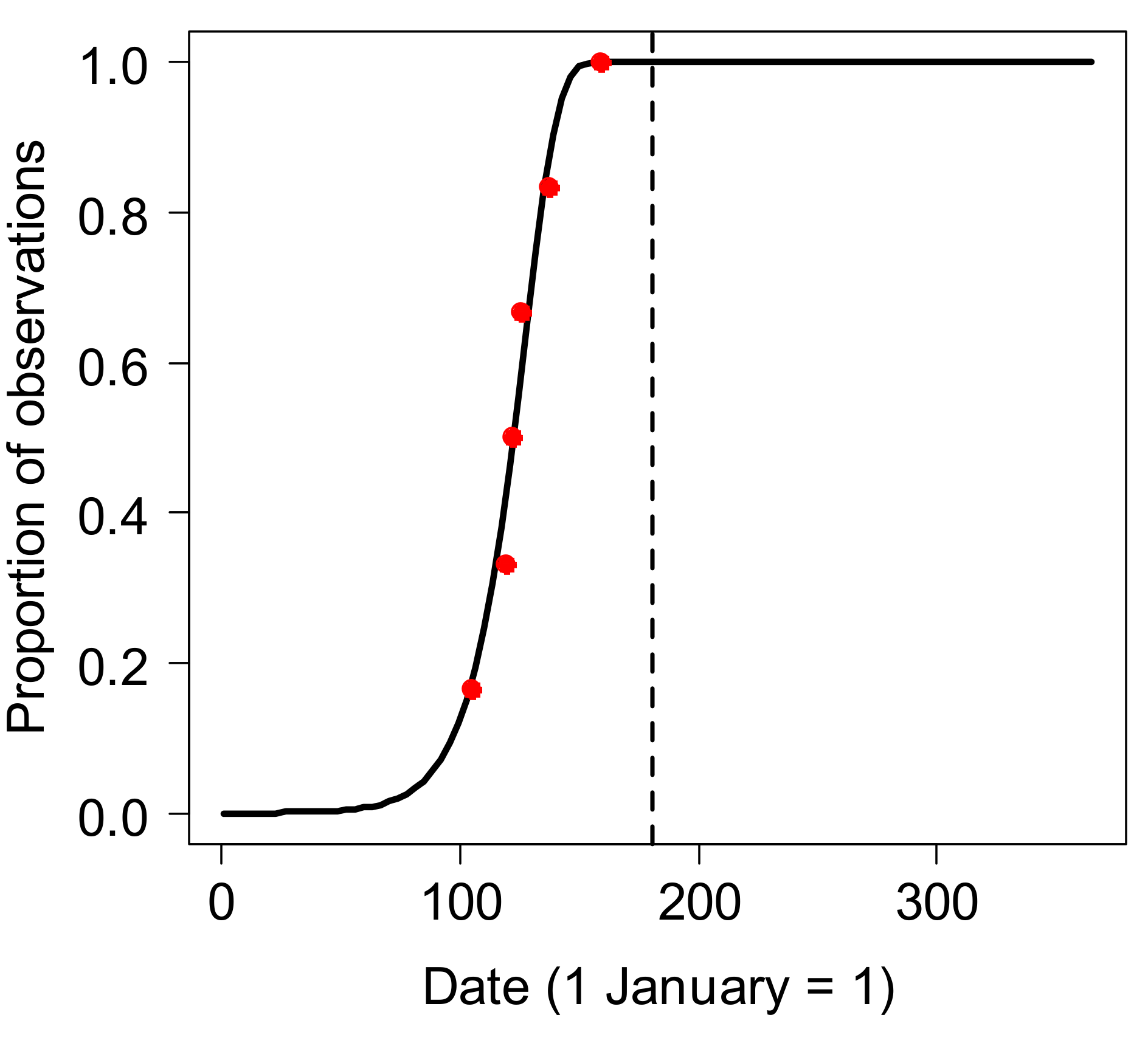

Supplement: Figure S2 — Example of data interpolation. Complementary log-log curve was interpolated to the March-June data from cell E1 in the British Isles (see Figure S1). Dots represent cumulated proportion of Barn Swallows recovered in this cell at different dates. All data in 1908–2008 were used. The dashed line represents June 30. (TIFF) [file pone.0102440.s002.tiff]

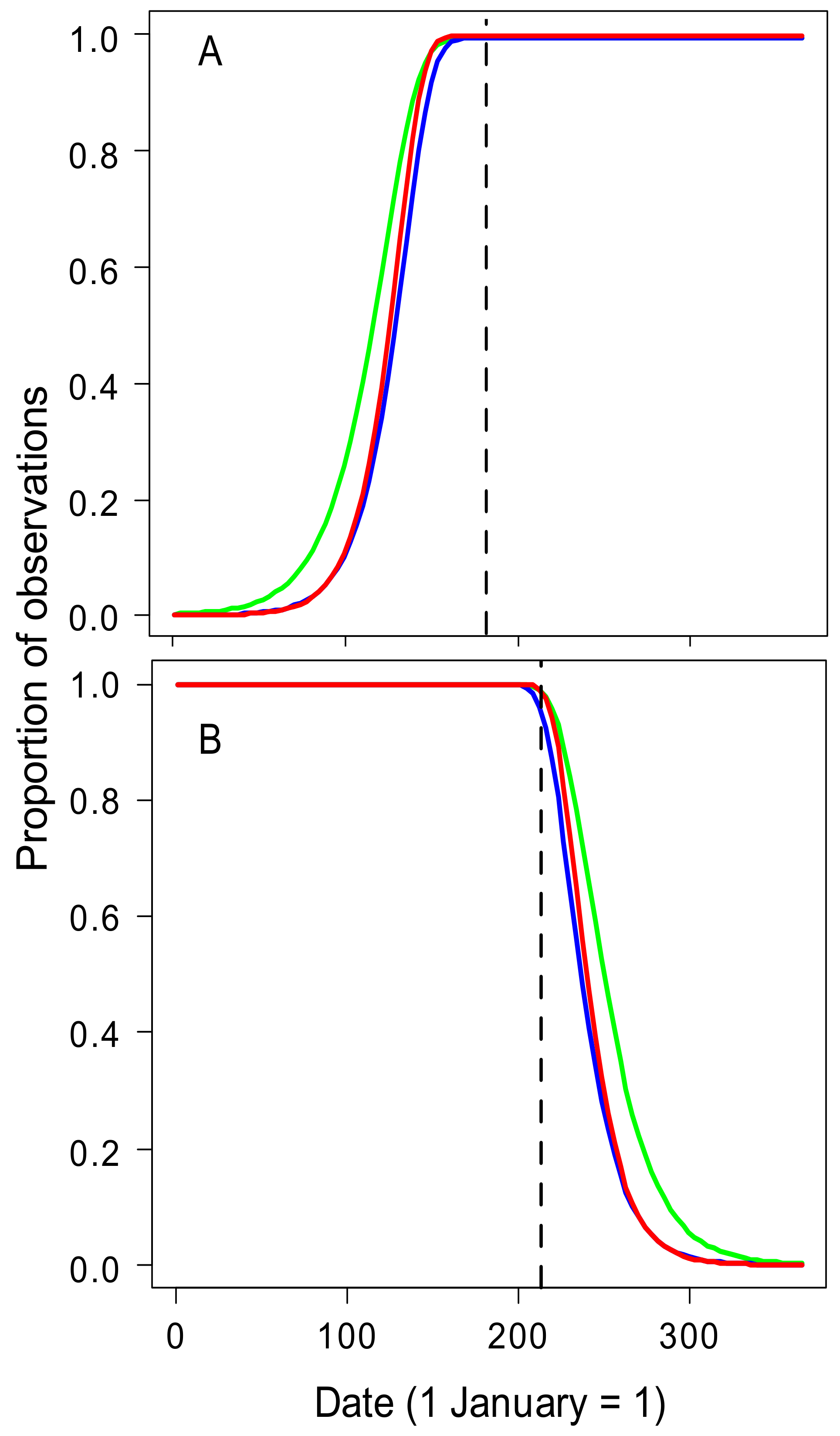

Supplement: Figure S3 — Interpolated curves showing shifts in migration phenology among periods. Complementary log-log curves were interpolated to cumulated proportions of Barn Swallows recovered during (A) February-June and (B) August-November during three different periods at cell G2 in western Europe and north Africa (Spain, see Figure S1). Green: curve fitted to data in 1908–1969; blue: curve fitted to data in 1970–1990; red: curve fitted to data in 1991–2008. In A) the delay in spring migration timing in 1970–1990 is evident as well as similarity in spring migration phenology pre-1970 and post-1990. Curves in (B) evidence the advancement in autumn migration phenology post-1970. The dashed line in (A) represents June 30 while that in (B) represents August 1. (TIFF) [file pone.0102440.s003.tiff]
