# Supplementary material for: Modelling the Progression of Bird Migration with Conditional Autoregressive Models Applied to Ringing Data
Source: PLoS One. 2014 Jul 21;9(7):e102440. doi: 10.1371/journal.pone.0102440 (PMC4105499; doi:10.1371/journal.pone.0102440)
Supplement: Table S1 — Comparison of observed and estimated phenology. General description of timing of migration and quantitative information from time-series of arrival dates of Barn Swallows in the British Isles and in western Europe and north Africa were collected from the literature, websites or other unpublished datasets, and compared with the corresponding estimate from our models. (PDF) [file pone.0102440.s004.pdf]

Table S1. Comparison of observed and estimated phenology. General description of timing of migration and quantitative information from time-series of arrival dates of Barn Swallows in the British Isles and in Europe were collected from the literature, websites or other unpublished datasets, and compared with the corresponding estimate from our models.

		Qualitative / quantitative description				Estimate from our model		
**Migration**	**Prog**	**Area – locality – type of description [reference]**	**Description**	**Interpretation**	**(Mean) Estimated date**	**Cell ID(s)**	**Percentage and (range of) predicted dates (min – max among cells)**	**(Mean) Estimated date**
Spring	1	North Africa and the Mediterranean – qualitative description [1]	Peak movement mid-March to late April	7 April	97	G1-G4, G6-G7, H1-H10**	50%: 10 April to 11 June	121
	2	North-west Europe – qualitative description [1]	Peak movement Mid-April to mid-May	30 April	120	B2, C1-C3, D1-D4**	50%: 28 May to 14 June	152
	3	Scotland – qualitative description [1]	First arrival in mid to late April	23 April	113	A4, B3-B5, C3-C5*	5%: 20 April to 13 May	117

4		Main flux in the first two weeks of May	7 May	127		50%: 28 May to 5 June	153
5	England – Norfolk – time series [2]	First arrival: 24 April	24 April	114	E7*	5%: 25 April	115
6	Northern Italy – Brescia – time series [3]	First arrival: 21 March	21 March	80	F5**	5%: 14 April	104
7	Northern Germany – Parchim – time series [4]	First arrival: 14 April	14 April	104	D6**	5%: 5 May	125
8	Southern Italy – Ventotene – time series [5]	Median trapping date 27-30 April (males-females)	29 April	119	G7**	50%: 24 April	115
9	Denmark – Kraghede – frequency of arrivals [6]	15%: 5 to 10 May	8 May	128	C5**	15%: 29 May	149
10		50%: 10 to 15 May	13 May	133		50%:15 June	166
11		85%: 20 to 25 May	23 May	143		85%: 26 June	177
12	Northern Italy - Varese – time series [our	First arrival: 26 march	26 March	85	F5**	5%: 14 April	104

		unpublished data]						
	13	Portland [7][§]	First arrival: 29 March	29 March	88	G5*	5%: 1 June	104
	14	Skokholm [7][§]	First arrival: 5 April	5 April	95	F3*	5%: 25 May	105
	15	Calf of Man [7][§]	First arrival: 9 April	9 April	99	D3*	5%: 3 June	112
	16	Hampshire [8][§]	First arrival: 23 March	23 March	82	F5, G5*	5%: 14 to 16 April	105
	17	West Midlands [9][§]	First arrival: 1 April	1 April	91	E5*	5%: 22 April	112
	18	Leicestershire [10][§]	First arrival: 20 March	20 March	79	E5-E6*	5%: 20 to 22 April	111
	19	Lancashire [11] [§]	First Arrival: 27 March	27 March	86	D4-D5, E4-E5*	5%: 18 to 22 April	110
Autumn	20	Scotland and Scandinavia – qualitative description [1]	Main migration in late August-September	10 September	253	B2, B4-B7, C2-C3, C6-C7**	50%: 12 to 28 August	235
	21	Southern Europe – qualitative description [1]	Main migration in September-early October	20 September	263	G1-G4, G6-G8, H7**	50%: 11 August to 8 October	251

	22	Spain – time series [12]	Mean departure date of last individual: 21 September	21 September	264	F2, G2-G3**	5% still remaining: 30 September to 31 October	285
	23	Col de Bretolet [13]	Mean Passage date: 20 September	20 September	263	E5**	50%: 1 September	244
	24	Northern Italy - Varese – time series [our unpublished data]	Mean departure date of last individual: 20 September	20 September	263	F5**	5% still remaining: 1 October	274
	25	Hampshire [8]	Latest departure date: 23 March	27 November	331	F5, G5*	5% still remaining: 25 September to 4 October	273
	26	West Midlands [9]	Latest departure date: 1 April	4 November	308	E5*	5% still remaining: 1 October	274
	27	Leicestershire [10][#]	Latest departure date: 20 March	2 November	306	E5-E6*	5% still remaining: 1 to 5 October	276
	28	Lancashire [11][#]	Latest departure date: 27 March	13 November	317	D4-D5, E4-E5*	5% still remaining: 21 September to 1 October	270

* Cell ID as reported in Figure Figure S1A or S1B

** Cell ID as reported in Figure Figure S1C or S1D

[§] see also: http://www.bto.org/volunteer-surveys/birdtrack/bird-recording/by-migration-season/spring-migration

[#]see also: http://www.bto.org/volunteer-surveys/birdtrack/bird-recording/by-migration-season/autumn-migration

References

[1] Turner A (2006) The Barn Swallow. London: T & A D Poyser. 256 p.

[2] Sparks TH, Carey PD (1995) The response of species to climate over two centuries: an analysis of the Marsham phenological record, 1736-1947. J Ecol 83: 321-329.

[3] Rubolini D, Ambrosini R, Caffi M, Brichetti P, Armiraglio S et al. (2007) Long-term trends in first arrival and first egg laying dates of some migrant and resident bird species in northern Italy. Int J Biometeorol 51: 553–563.

[4] Schmidt E, Hüppop K (2007) First observation and start of birdsong of 97 bird species in a community in the county of Parchim (Mecklenburg-Vorpommern) in the years 1963 to 2006. Vogelwarte 45: 27–58.

[5] Spina F, Massi A, Montemaggiori A (1994) Back from Arica: who's running ahead? Differential migration of sex and age classes in Palearctic-African spring migrants. Ostrich 65: 137-150.

[6] Møller AP (1994) Phenotype-dependent arrival time and its consequences in a migratory bird. Behav Ecol Sociobiol 35: 115-122.

[7] Loxton D, Sparks T (1999) Arrival of spring migrants at Portland, Skokholm, Bardsey and Calf of Man: Bardsey Observatory Report 42: 1998.

[8] Cox A (2002) Hampshire Bird Report 2002. Chandler's Ford: Hampshire Ornithological Society.

[9] Emley DW (2003) The Birds of Staffordshire, Warwickshire, Worcestershire and the West Midlands 2001 Annual Report 68. Studley: West Midland Bird Club.

[10] Mackay AJ (2002) The Leicestershire and Rutland Bird Report 2001. Leicester: The Leicestershire and Rutland Ornithological Society.

[11] White SJ (2003) Lancashire Bird Report 2002. Preston: Lancashire & Cheshire Fauna Society No 104.

[12] Gordo O, Sanz JJ (2006) Climate change and bird phenology: a long-term study in the Iberian Peninsula. Glob Change Biol 12: 1993-2004.

[13] Jenni L, Kéry M (2003) Timing of autumn bird migration under climate change: advances in long-distance migrants, delays in short-distance migrants. Proc R Soc Lond B 270: 1467-1471.
