# Supplementary material for: Modelling the Progression of Bird Migration with Conditional Autoregressive Models Applied to Ringing Data
Source: PLoS One. 2014 Jul 21;9(7):e102440. doi: 10.1371/journal.pone.0102440 (PMC4105499; doi:10.1371/journal.pone.0102440)
Supplement: Text S1 — The complementary log-log and logistic functions. A brief description of complementary log-log and logistic functions. (PDF) [file pone.0102440.s005.pdf]

SUPPORTING INFORMATION FOR

Modelling the Progression of Bird Migration with Conditional Autoregressive Models Applied to Ringing Data

Roberto Ambrosini[1*], Riccardo Borgoni[2], Diego Rubolini[3], Beatrice Sicurella[1], Wolfgang Fiedler[4], Franz Bairlein[5], Stephen R. Baillie[6], Robert A. Robinson[6], Jacquie A. Clark[6], Fernando Spina[7], Nicola Saino[3]

1 Dipartimento di Biotecnologie e Bioscienze, Università degli Studi di Milano-Bicocca, Milano, Italy

2 Dipartimento di Economia, Metodi quantitativi e Strategie di Impresa, Università degli Studi di Milano-Bicocca, Milano, Italy

3 Dipartimento di Bioscienze, Università degli Studi di Milano, Milano, Italy

4 Max Plank Institute for Ornithology, Vogelwarte Radolfzell, Radolfzell, Germany

5 Institute of Avian Research "Vogelwarte Helgoland", Wilhelmshaven, Germany

6 British Trust for Ornithology, Thetford, UK

7 Istituto Superiore per la Protezione e la Ricerca Ambientale, Ozzano dell'Emilia (BO), Italy

*Correspondence author: Roberto Ambrosini, Dipartimento di Biotecnologie e Bioscienze, Università degli Studi di Milano-Bicocca, piazza della Scienza 2, 20126 Milano, Italy. Email: roberto.ambrosini@unimib.it

The Complementary Log-Log and the Logistic Function

Binomial models can be fitted by choosing different link functions [1]. Taking our dataset as an example, the link function η defines the relationship between the expected values of the proportion p_t of swallows observed at a cell until a given calendar date t and date itself. Formally

$$\eta(E(p_t)) = \alpha + \beta t \qquad \text{Eq. 1}$$

The common choice for η is the logit function:

$$\eta(p) = \log \frac{p}{1-p} \qquad \text{Eq. 2}$$

By substituting Eq. 2 into Eq 1 with p = $E(p_t)$ and then solving for $E(p_t)$ we obtain

$$E(p_t) = \frac{e^{\alpha+\beta t}}{1+e^{\alpha+\beta t}} \qquad \text{Eq. 3}$$

Eq. 3 is the logistic function (i.e. the inverse of the logit function) and can be used to calculate the expected proportion of arrivals until a given calendar date from the coefficient of a binomial model fitted with a logistic link function.

Binomial models can also be fitted by using alternative link functions, that can be a better choice than the logistic function in particular circumstances. In particular, Baddeley *et al.* [2] demonstrated that when spatial Poisson processes are analysed by dividing the space into pixels, the optimal estimator of the probability of occurrence (or, equally, of the proportion of occurrences) in a pixel is the complementary log-log (‘cloglog’) function:

$$\eta(p) = \log(-\log(1-p)) \qquad \text{Eq. 4}$$

The inverse of Eq. 4 is

$$E(p_t) = 1 - e^{-e^{\alpha+\beta t}} \qquad \text{Eq. 5}$$

We can solve Eq. 5 for t obtaining

$$t = (\log(-\log(1-p)) - \alpha)/\beta \qquad \text{Eq. 6}$$

Eq. 6 allows estimating the calendar date at which a given proportion p of swallows is expected to have been observed based on the parameters α and β of the cloglog curve.

The logistic function is symmetric about p = 0.5, while the cloglog is not, having a longer tail at the lower end (Figure R1). Both functions can be fitted to the same data by specifying the opportune link function in the binomial GLM or GLMM.

Figure R1. Logistic (blue) and complementary log-log (red) curves interpolated to spring migration data in western Europe and north Africa. Parameters of the model were estimated by binomial CAR GLMMs as specified in the main text. Both models were fitted with the *lmer* procedure in the *lme4* package [3] in R 2.15.2 [4] by specifying respectively the logit and the cloglog link functions.

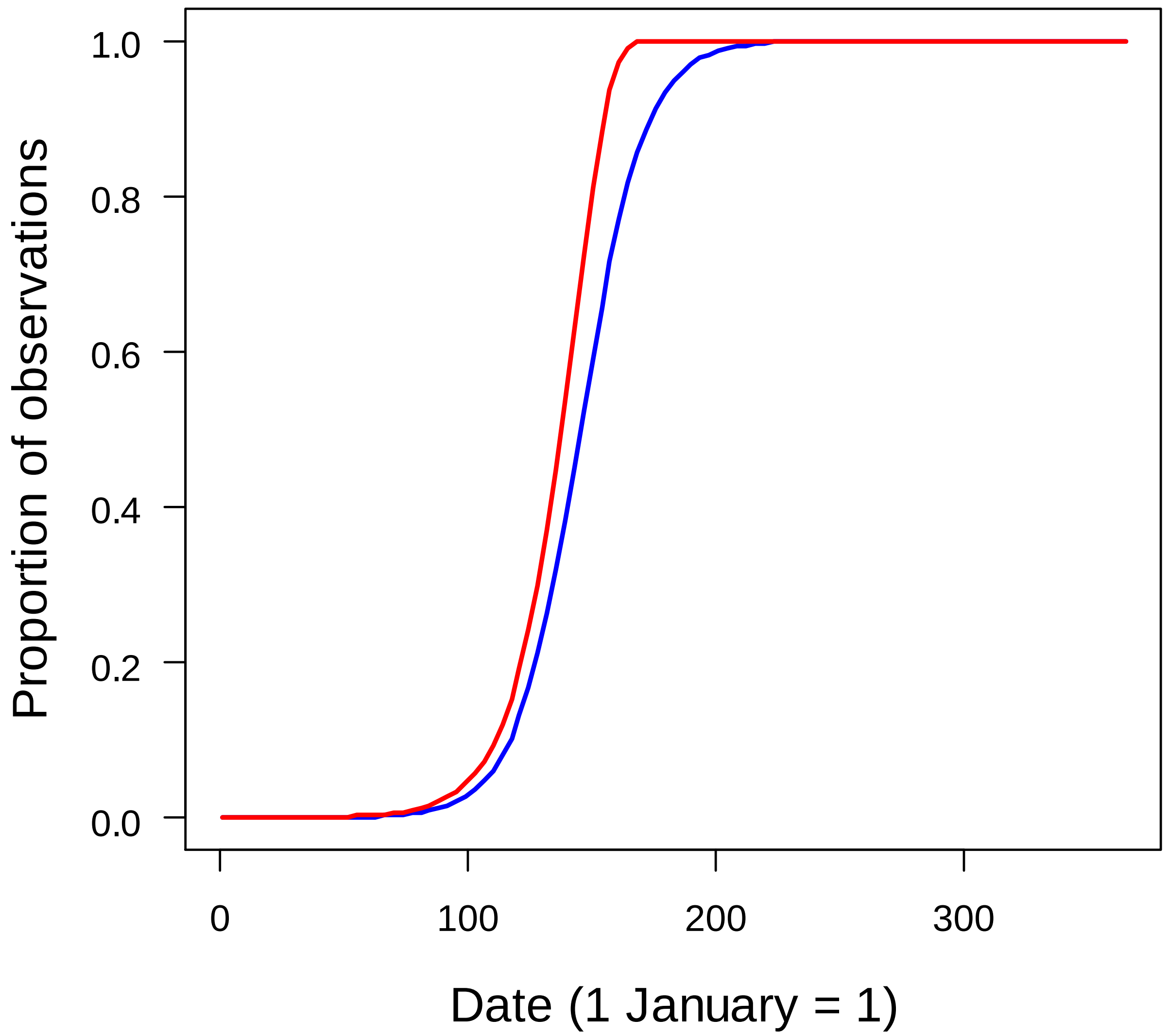

References

[1] Zuur AF, Ieno EI, Walker NJ, Saveliev AA, Smith GM (2009) Mixed effects models and extensions in ecology with R. New York: Springer. 574 p.

[2] Baddeley A, Berman M, Fisher NI, Hardegen A, Milner RK et al. (2010) Spatial logistic regression and change-of-support in Poisson point processes. Electron J Statist 4: 1151-1201.

[3] Bates D, Maechler M, Bolker B (2012) *lme4*: linear mixed-effects models using S4 classes. R package version 0.999999-0.

[4] R Core Team (2012) R: a language and environment for statistical computing. Vienna: R Foundation for Statistical Computing. 1731 p.
